# Depression and Life Satisfaction Among Middle-Aged and Older Adults: Mediation Effect of Functional Disability

**DOI:** 10.3389/fpsyg.2021.755220

**Published:** 2021-11-25

**Authors:** An Li, Dewen Wang, Shengnan Lin, Meijie Chu, Shiling Huang, Chun-Yang Lee, Yi-Chen Chiang

**Affiliations:** ^1^State Key Laboratory of Molecular Vaccinology and Molecular Diagnostics, School of Public Health, Xiamen University, Xiamen, China; ^2^School of Public Affairs, Xiamen University, Xiamen, China; ^3^School of International Business, Xiamen University Tan Kah Kee College, Zhangzhou, China

**Keywords:** functional disability, depression, life satisfaction, mediator, gender

## Abstract

With increasing age, middle-aged and older persons face a series of physical and mental health problems. This study aimed to explore the latent relationships among age, functional disability, depression, and life satisfaction. The data were obtained from the Wave 2 (in 2013–2014) and Wave 3 (in 2015–2016) surveys of the China Health and Retirement Longitudinal Study. The analytic sample in the present study included 15,950 individuals aged 45 years and over. The participants answered the same questions concerning depression and life satisfaction in both study waves, and functional disability was measured based on the activities of daily living and instrumental activities of daily living. Age was directly associated with functional disability, life satisfaction, and depression. Functional disability was positively correlated with depression and negatively correlated with life satisfaction. Functional disability strongly mediated the relationships among age, depression, and life satisfaction. Depression and life satisfaction were found to have enduring effects and effects on each other. Additionally, the model revealed a gender difference. Depression in middle-aged people should receive closer attention. Avoiding or improving functional disability may be an effective way to improve life satisfaction and reduce the level of depression in middle-aged and older persons. If prevention work successfully decreases depression, the life dissatisfaction of middle-aged and older people could be improved. Additionally, for the prevention of functional disability and depression and improvement in life satisfaction, gender differences need to be considered.

## Introduction

The growth rate of the aging population is unprecedented worldwide and will accelerate in the coming decades, especially in developing countries (WHO, 2021a). There are 1 billion people aged 60 and over worldwide. This number is estimated to reach 1.4 billion by 2030 and 2.1 billion by 2050 (WHO, 2021a). As a developing country with the largest population worldwide, China was home to 176 million older persons aged 65 years old and over (12.57% of the country’s population) in 2019. By 2050, China’s older population (65+) is likely to rise to 330 million, representing approximately a quarter of the population (Wang et al., 2016). As human life expectancy increases, older people are likely to experience reduced physical capacity (Auais et al., 2019), adding to the associated burden (Song et al., 2017). The early prevention of age-related diseases can reduce the negative effects of aging and the disease burden on the older population (Partridge et al., 2018). Therefore, while addressing the health problems of older people, prevention at a young age should also be emphasized to promote healthy aging.

Maintaining the ability to move and function independently in old age are critical for continued community participation, health, and well-being and is a major challenge posed by population aging (Auais et al., 2019). With increasing age, an increasing number of middle-aged and older people can suffer from functional disabilities that affect their mobility, social participation, and quality of life (Webber et al., 2010; Sole-Auro and Alcaniz, 2015). Research has shown that age is one of the main reasons for the impairment of physical ability among older persons (Falk et al., 2014). As people get older, skeletal muscle degenerates, muscle mass and muscle strength gradually decrease, and functional performance decreases (Barberi et al., 2015).

Functional disability is defined as the need for help or the inability to perform one or more activities of daily living (ADL) or instrumental activities of daily living (IADL) (Griffith et al., 2010). ADL refer to the most basic and common body movements that people must perform repeatedly daily to live independently, such as bathing, eating, dressing, getting up and down from bed, going to the toilet, and controlling urine and feces, reflecting the most basic self-care ability. ADL are important indices for predicting the life span and determining the quality of life (Covinsky, 2006). IADL refer to the adaptive work that individuals perform to cope with the needs of their environment. IADL are often complex and require good abilities to perform, such as shopping, cooking, completing household chores, doing the laundry, using the telephone, and managing money. These activities, while not necessary every day, are important for maintaining an individual’s independence. Since ADL measures do not measure the ability of older people to adapt to the environment, they underestimate the number of older people who need assistance in various living activities. Therefore, the inclusion of both ADL and IADL items could better determine the extent of community dysfunction and identify broader service needs (Spector et al., 1987).

Based on the World Health Organization (WHO) definition, health is a state of complete physical, mental, and social well-being and not merely the absence of disease or infirmity (Kühn and Rieger, 2017). In recent years, mental health has played an important role in achieving global development goals, as illustrated by the inclusion of mental health in the 2030 agenda for sustainable development (UN, 2015). When one’s physical health worsens, control over activities is restricted, which endangers mental health (Rodin, 1986). Therefore, attention should be given to both mental health and physical health.

As two important dimensions of mental health, depression and life satisfaction need to be considered (Headey et al., 1993; Guney et al., 2010). Depression is a non-communicable disease with a significant global disease burden (Hay et al., 2017). As a common mental illness (Liu Q. et al., 2020), depression affects approximately 264 million people worldwide (WHO, 2021b). In China, depression is also a common disease and is a major public health challenge requiring urgent prevention. A systematic analysis showed that 2.2% of men and 3.3% of women suffer from major depression in China (Baxter et al., 2016). A nationally representative China Longitudinal Aging Social Survey also confirmed that depression was higher in women than men (Li and Chen, 2021). Depression not only affects work (Jantaratnotai et al., 2017) and increases the economic burden on society and families (Kordy et al., 2013), but also increases the risk of suicidal ideation and behavior (Teng et al., 2013). Therefore, it is very important to identify the risk factors for depression and implement effective intervention strategies to prevent or delay the development of depression. Depression has a U-shaped overall distribution in the population, but as far as the middle-aged and elderly people are concerned, the incidence of depression in older people is higher than that in middle-aged people (Mirowsky and Ross, 1992). The closer the older adults are to the end of life, the more they experience stressful events, such as illness, declining income, and the death of relatives and friends. The life cycle hypothesis holds that the average level of depression declines during early adulthood to middle age and then rises (Mirowsky and Ross, 1992). Therefore, it is generally believed that with an increase in age, depression among middle-aged and older adults will continue to increase (Bergdahl et al., 2005; Solhaug et al., 2012). However, a few studies have found that depression among older people decreases with age (Sung, 2013). Therefore, the effect of age on depression needs to be further verified.

In contrast to depression, life satisfaction is a positive evaluation index; it is a subjective well-being measure reflecting a person’s cognitive judgment of life (Diener, 1984; Hong et al., 2019). Life satisfaction is an important goal for improving the quality of life of older adults (Chachamovich et al., 2007), and it is an indispensable cognitive or evaluative element of life quality and successful aging (Lawton et al., 2002). However, life satisfaction among middle-aged and older adults is related to many factors, including age. What is the relationship between age and life satisfaction? Some studies have shown a U-shaped relationship between age and life satisfaction (Blanchflower and Oswald, 2004; Graham and Pozuelo, 2017), meaning that life satisfaction declines in middle age and then increases with age. In other words, starting in middle age, people’s life satisfaction increases with age. According to socioemotional selectivity theory (SST), future time perspective (FTP) is a key factor in explaining the persistent or even improved subjective well-being of elderly individuals compared with that of their younger counterparts (Carstensen et al., 1999; Carstensen, 2006). With limited FTP, older people tend to focus on the present rather than the future, which benefits their subjective well-being. However, contrary to the assertion of SST, many studies have found that persons with a limited FTP tend to report lower subjective well-being (Allemand et al., 2012; Kozik et al., 2015; Gruhn et al., 2016). According to life span theories of motivation (Heckhausen, 2000; Hong et al., 2019), young and middle-aged people focus on growth and self-development, while older people are increasingly aware of the decline in biological function and the limited resources and opportunities in the future (Cheng et al., 2009). Their motivation focuses on maintaining the current level of function and planning for future decline. Thus, the youngest adults show positive trajectories in terms of perceived past, present, and future life satisfaction, while the trajectory is flat in late middle age and negative in older adults. This result has also been verified (Hong et al., 2019).

The research shows that in addition to age, functional disability is an important factor influencing depression and life satisfaction among middle-aged and older people (Liang et al., 2017; Barry et al., 2020). According to the stress process theory (Pearlin et al., 1981), dysfunction may hinder people’s ability to achieve their expected social role of living independently, ultimately leading to depression (Russell et al., 2009). Studies have shown that dysfunction in IADL/ADL leads to a significant increase in depression among older persons in general (Bozo et al., 2009; Park et al., 2014). The research (Peltzer and Phaswana-Mafuya, 2013) examining the factors associated with depression among South Africa adults revealed that functional disability was significantly associated with increased depression. Additionally, according to a follow-up study in China, ADL disability could increase the risk of depressive symptoms in middle-aged and older adults and their spouses (He et al., 2019). Compared with non-disabled people, older people with disabilities have higher levels of depression (Pagan-Rodriguez and Perez, 2012).

The relationship between depression and life satisfaction in middle-aged and older adults has also been discussed. One study found that depression has the greatest influence on older adults’ life satisfaction (Sok, 2010), while another study reported that life satisfaction is the strongest negative predictor of depression in older adults (Yoo et al., 2016). The possible pathways have yet to be determined through structural equation modeling (SEM). In summary, the relationship among age, functional disability, depression, and life satisfaction needs to be further verified among middle-aged and older adults using national survey data.

**FIGURE 1:**
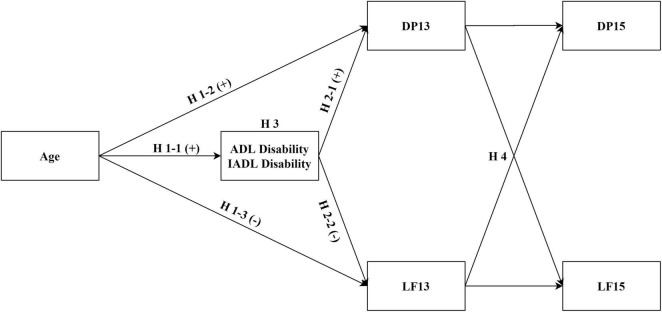
Hypothesized model of the research framework. ADL, activities of daily living; IADL, instrumental activities of daily living; DP13, depression in 2013–2014; DP15, depression in 2015–2016; LF13, life satisfaction in 2013–2014; LF15, life satisfaction in 2015–2016. Hypotheses were that (H1-1) age would be positively correlated with functional disability; (H1-2) age would be positively correlated with depression; (H1-3) age would be negatively correlated with life satisfaction; (H2-1) functional disability would be positively correlated with depression; (H2-2) functional disability would be negatively correlated with life satisfaction; (H3) functional disability would mediate the relationships of age with depression and life satisfaction; and (H4) there would be a cross effect between depression and life satisfaction.

What is the relationship among age, depression, and life satisfaction among middle-aged and older adults? Is functional disability a mediator between age and depression/life satisfaction? Based on the existing literature and theories, our hypotheses (as shown in Figure 1) are as follows: among middle-aged and older adults (H1-1), age is positively correlated with functional disability (ADL disability and IADL disability); (H1-2) age is positively correlated with depression; (H1-3) age is negatively correlated with life satisfaction; (H2-1) functional disability is positively correlated with depression; (H2-2) functional disability is negatively correlated with life satisfaction; (H3) functional disability mediates the relationships between age and depression or life satisfaction; and (H4) a cross effect exists between depression and life satisfaction.

## Materials and Methods

### Participants

The data were obtained from the Wave 2 (in 2013–2014) and Wave 3 (in 2015–2016) surveys of the China Health and Retirement Longitudinal Study (CHARLS), which is an ongoing nationwide population-based prospective cohort study (Zhao et al., 2014). The sample is representative of the household population aged 45 years and older in China (baseline survey in 2011–2012, with participants recruited from 450 villages and residences in 150 counties and districts in 28 provinces). The follow-up time interval of the CHARLS is 2 years. Therefore, the CHARLS participants received the Wave 2 survey in 2013–2014 and the Wave 3 survey in 2015–2016. For more details on the recruitment strategy, design, and sampling method of the CHARLS, refer Zhao et al. (2014).

**FIGURE 2:**
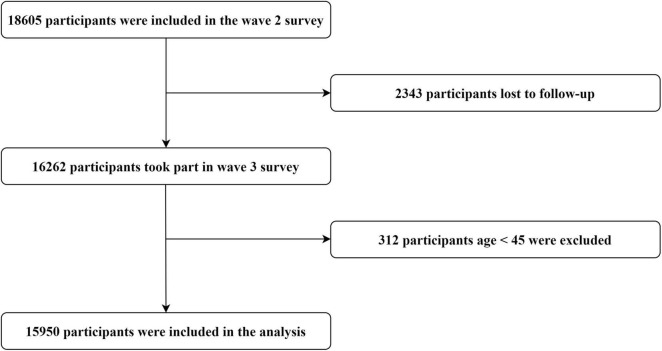
Flowchart of the inclusion and exclusion of participants.

A flowchart of the sample selection in this study is shown in Figure 2. First, in total, 18,605 respondents participated in the Wave 2 survey. Then, 2343 participants were excluded because they did not continue to participate in the Wave 3 survey. Since the survey involved people aged over 45 years, 312 participants younger than 45 were further excluded. Finally, in total, 15,950 participants were included in our analysis. The follow-up rate was over 85%. Consequently, in the final sample, 7627 men (47.83%) and 8319 women (52.17%) responded to the two interviews. To prove that the data are representative, we conducted attrition analysis of ADL disability, IADL disability, depression, life satisfaction, gender, and age. Since ADL disability, IADL disability, and depression were continuous variables, and life satisfaction was an ordinal variable, we used the values of percentile 25 and percentile 75 of each variable (ADL disability, IADL disability, and depression) in the first wave to divide the whole sample into three groups, and life satisfaction was divided into five groups according to its options. Then Chi-square goodness-of-fit test was used to conduct the attrition analysis (compared the distribution of variables among the second-wave follow-up data and the first wave data). The results showed that there was no statistically significant difference in attrition of IADL disability (χ^2^ = 2.48, *p* = 0.29), depression (χ^2^ = 0.09, *p* = 0.95), life satisfaction (χ^2^ = 1.89, *p* = 0.76), and gender (χ^2^ = 0.12, *p* = 0.72), except for ADL disability and age. But this may be due to excessive sensitivity resulting from the large sample size. In addition, descriptive analysis found that the ADL disability and age distribution difference caused by attrition was only 0.39–1.09 and 1.11–2.37%, respectively. The CHARLS program was approved by the Ethical Review Committee of Peking University, and all participants signed an informed consent form.

### Measures

#### Activities of Daily Living and Instrumental Activities of Daily Living

Difficulty with ADL in the past 3 months was measured using the Barthel Index for ADL (Wade and Collin, 1988; Liu N. et al., 2020). ADL information was collected with six questions about difficulty in dressing, bathing, eating, getting into or out of bed, using the toilet, and controlling urination and defecation. Respondents were also asked whether they had any difficulty performing IADL (Lawton and Brody, 1969; Liu N. et al., 2020), including doing household chores, preparing hot meals, shopping for groceries, making phone calls, taking medications, or managing money, in the past 3 months. Each item was scored from 1 (“don’t have any difficulty”) to 4 (“can’t do it”) points, with the highest score indicating the greatest symptom burden. The same questions were used in both Wave 2 and Wave 3 of the data collection but not in Wave 1 (the question regarding phone calls was not asked in this survey). In this study, to verify the effects of ADL disability and IADL disability on depression and life satisfaction, we used the functional disability data from Wave 2. The Cronbach’s alphas for the ADL and IADL disability measures were 0.85 and 0.83, respectively.

#### Depressive Symptoms

The participants self-reported their depressive symptoms using The Center for Epidemiological Studies Depression Scale (CES-D) short form (Kohout et al., 1993; Wang et al., 2019; Qiao et al., 2021) at baseline and each follow-up of the CHARLS. The CES-D short form is composed of 10 items, which is used to evaluate the frequency of symptoms or behaviors experienced during the past week, such as “I was bothered by things that don’t usually bother me,” “I felt depressed,” and “I felt fearful.” Question 5 (“I felt hopeful about the future”) and Question 8 (“I was happy”) are reverse questions, and the responses were reverse-scored before analysis. Each item is scored on a scale from 1 to 4, where 1 = “rarely or none of the time,” 2 = “some or a little of the time,” 3 = “occasionally or a moderate amount of the time,” and 4 = “most or all of the time.” Higher scores indicate more severe depressive symptoms. Previous research has shown that the CES-D short form is reliable and valid (Lei et al., 2014). In our data, the Cronbach’s alphas for this measure were 0.78 (Wave 2) and 0.81 (Wave 3).

#### Life Satisfaction

In both Wave 2 and Wave 3, life satisfaction was measured by asking participants “Please think about your life as a whole. How satisfied are you with it?” The five possible responses ranged from “completely satisfied” to “not at all satisfied.” The responses were reverse-scored before analysis.

#### Data Analyses

Statistical analyses were performed using SAS version 9.4 (Copyright © SAS Institute Inc., SAS Campus Drive, Cary, North Carolina 27513, United States. All rights reserved), LISREL version 8.80 (Copyright 2006, Scientific Software International Inc., All rights reserved), IBM SPSS STATISTICS 22.0 (SPSS Inc., Chicago, IL, United States), and R version 4.0.2 (R Foundation, Vienna, Austria). SAS was used to merge the Wave 2 and Wave 3 data based on individual IDs, SPSS was used to analyze the correlations between the variables. Cronbach’s alpha coefficient was used to evaluate the internal consistency of the scales. Confirmatory factor analysis (CFA) was used to test the construct validity of the scales (Table 1). The goodness-of-fit indices for the CES-D short-form were as follows: (1) root mean squared error of approximation (RMSEA) = 0.057; (2) comparative fit index (CFI) = 0.98; and (3) tucker-lewis index (TLI) = 0.98. The goodness-of-fit indices of the ADL and IADL disability measures were as follows: (1) RMSEA = 0.041; (2) CFI = 1; and (3) TLI = 1. If CFI > 0.92, or RMSEA < 0.07, the model is considered to fit the data (Hair et al., 2014; Mora-Pelegrin et al., 2021). The results indicate that these instruments had good reliability and validity.

**TABLE 1:** Confirmatory factor analysis and structural equation modeling.

Variables	Items	Confirmatory factor analysis	
		λ	*t*
**Functional disability:** Because of health and memory problems, do you have any difficulty with……			
1. Activities of daily living (ADL)	Dressing?	0.97	111.85
	Bathing or showering?	1.00	–
	Eating, such as cutting up your food?	0.96	71.86
	Getting into or out of bed?	0.93	96.68
	Using the toilet, including getting up and down?	0.85	89.51
	Controlling urination and defecation?	0.73	40.20
2. Instrumental activities of daily living (IADL)	Doing household chores?	0.99	149.46
	Preparing hot meals?	0.99	146.40
	Shopping for groceries?	0.95	120.97
	Making phone calls?	0.65	44.14
	Taking medications?	0.81	55.39
	Managing your money, such as paying your bills, keeping track of expenses, or managing assets?	0.81	72.91
Depression (2013–2014)	I was bothered by things that don’t usually bother me.	0.84	76.43
	I had trouble keeping my mind on what I was doing.	0.77	65.22
	I felt depressed.	1.00	–
	I felt everything I did was an effort.	0.87	77.74
	I felt hopeful about the future.	0.22	15.78
	I felt fearful.	0.83	59.38
	My sleep was restless.	0.63	51.76
	I was happy.	0.48	38.73
	I felt lonely.	0.88	73.49
	I could not get “going.”	0.93	73.64

*Items of ADL and IADL disability scores 1 (don’t have any difficulty) to 4 (can’t do it) points; items of depression scores 1 (rarely or none of the time) to 4 (most or all of the time) points.*

To investigate the correlations among age, ADL disability, IADL disability, depression, and life satisfaction in middle-aged and older individuals, SEM was performed using LISREL version 8.80 (Copyright 2006, Scientific Software International Inc., All rights reserved.). The maximum likelihood estimation method was used. In addition, the estimated indirect effects (IEs) in the output file from LISREL and Monte Carlo resampling with R were used to confirm the significance of the IEs.

## Results

### Descriptive Statistics and Correlation Analysis

After the inclusion and exclusion criteria were applied, 15,950 older adults were included in the analytical sample. Table 2 shows the means, SDs, and correlation coefficients of all variables.

**TABLE 2:** Descriptive statistics and correlation analysis.

Variables	*M* ± SD	1	2	3	4	5	6	7
1. Age	60.04 ± 9.66	1						
2. ADL disability	6.66 ± 1.78	0.17***	1					
3. IADL disability	7.13 ± 2.77	0.23***	0.04***	1				
4. DP13	17.84 ± 5.77	−0.05***	0.31***	0.16***	1			
5. DP15	18.14 ± 6.46	–0.04	0.20***	0.10***	0.61***	1		
6. LF13	3.12 ± 0.74	0.13***	−0.11***	−0.04***	−0.06***	−0.10***	1	
7. LF15	3.39 ± 0.78	0.05***	−0.09***	−0.04***	−0.20***	−0.31***	0.32***	1

M, mean; ADL, activities of daily living; IADL, instrumental activities of daily living; DP13, depression in 2013–2014; DP15, depression in 2015–2016; LF13, life satisfaction in 2013–2014; LF15, life satisfaction in 2015–2016. ***p < 0.001.

The results showed that IADL disability and ADL disability were positively correlated with depression in 2013–2014 (DP13) and depression in 2015–2016 (DP15) and negatively correlated with life satisfaction in 2013–2014 (LF13) and life satisfaction in 2015–2016 (LF15). There was also a negative correlation between depression and life satisfaction in both surveys. A *t*-test revealed that there were significant gender differences in IADL disability (*t* = −10.28, *p* < 0.01), DP13 (*t* = −18.32, *p* < 0.001), and DP15 (*t* = −21.07, *p* < 0.001). The Chi-square test also showed significant gender differences in LF13 (*p* < 0.05) and LF15 (*p* < 0.05). Compared to men, women had higher levels of IADL disability and depression and lower life satisfaction. There was no significant gender difference in ADL disability.

### Direct/Indirect Effect Analyses

**FIGURE 3:**
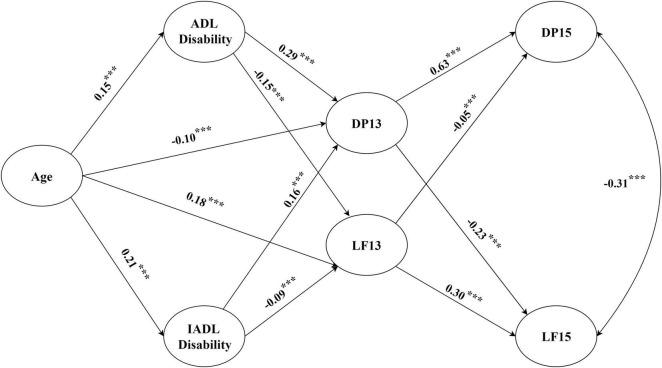
Structural equation modeling. The model presented the direct and indirect effects between variables. Gender, as a control variable, is not shown in the figure. ADL, activities of daily living; IADL, instrumental activities of daily living; DP13, depression in 2013–2014; DP15, depression in 2015–2016; LF13, life satisfaction in 2013–2014; LF15, life satisfaction in 2015–2016. ***p < 0.001.

Based on the preset model, ADL disability and IADL disability were added to the structural equation model as mediating variables in the direct path from age to depression and life satisfaction, and LF15 was set to be correlated with DP15 after controlling for sex (Figure 3). The results suggested a good fit of the data to the model (RMSEA = 0.033; CFI = 0.99; TLI = 0.99).

The SEM showed that after ADL disability and IADL disability were added as mediation variables, there was a significant negative correlation between age and DP13 (β = −0.10, *p* < 0.001), and LF13 increased with age (β = 0.18, *p* < 0.001); this result differed from the hypothesis, so H1-2 and H1-3 were not supported. There was a significant positive correlation between age and ADL disability (β = 0.15, *p* < 0.001)/IADL disability (β = 0.21, *p* < 0.001); thus, H1-1 was supported.

Regarding functional disability, ADL disability (β = 0.29, *p* < 0.001) and IADL disability (β = 0.16, *p* < 0.001) were positively correlated with DP13; in contrast, ADL disability (β = −0.15, *p* < 0.001) and IADL disability (β = −0.09, *p* < 0.001) were negatively correlated with LF13, supporting H2-1 and H2-2. Therefore, ADL disability and IADL disability partially mediated the effects of age on depression and life satisfaction, and H3 was preliminarily supported.

The DP13 was negatively correlated with LF15 (β = −0.23, *p* < 0.001), and LF13 was negatively correlated with DP15 (β = −0.05, *p* < 0.001), indicating that depression and life satisfaction may affect each other; thus, H4 was supported. DP13 was positively correlated with DP15 (β = 0.63, *p* < 0.001), and LF13 was positively correlated with LF15 (β = 0.30, *p* < 0.001), which indicates that depression and life satisfaction have enduring effects. In addition, we found that DP13 played a mediating role in the relationships of age, ADL disability, and IADL disability with LF15 and that LF13 played a mediating role in the relationships of age, ADL disability, and IADL disability with DP15.

To further verify the mediation hypotheses (H3), Monte Carlo resampling was used to construct the CIs (Preacher and Selig, 2012). Specifically, a program was written in R to construct 95% CIs for the IEs based on 20,000 resamples (Preacher and Selig, 2012). According to the results (Table 3), the 95% CI for all IEs did not include zero. Therefore, H3 was further supported.

**TABLE 3:** Tests of indirect effects of the hypothesized model by Monte Carlo approach of resampling (total sample, *n* = 15,950).

Path	Indirect effect	95% CI
Age→ADL disability→DP13	0.044	[0.037, 0.050]
Age→ADL disability→LF13	–0.023	[−0.032, −0.013]
Age→IADL disability→DP13	0.034	[0.026, 0.041]
Age→IADL disability→LF13	–0.019	[−0.030, −0.008]
Age→DP13→LF15	0.023	[0.017, 0.030]
Age→LF13→DP15	–0.009	[−0.013, −0.005]
ADL disability→DP13→LF15	–0.067	[−0.081, −0.053]
ADL disability→LF13→DP15	0.008	[0.004, 0.012]
IADL disability→LF13→DP15	0.005	[0.002, 0.008]
IADL disability→DP13→LF15	–0.037	[−0.047, −0.027]

*ADL, activities of daily living; IADL, instrumental activities of daily living; DP13, depression in 2013–2014; DP15, depression in 2015–2016; LF13, life satisfaction in 2013–2014; LF15, life satisfaction in 2015–2016.*

### Structural Equation Modeling Among Different Gender

**FIGURE 4:**
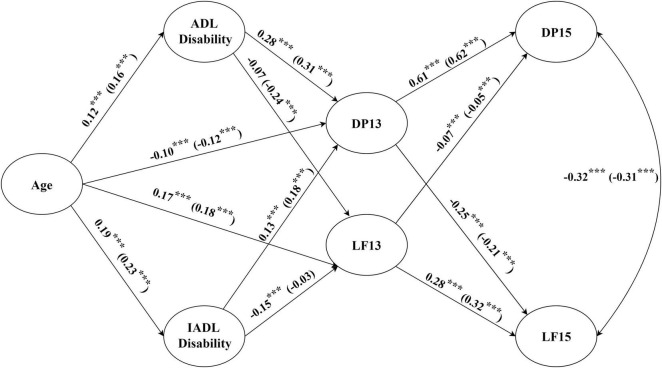
Structural equation modeling for different genders. The coefficients inside the brackets are for women. Significant paths with gender difference according to the multi-group comparison analysis by LISREL: Age→ADL disability (Δχ^2^ = 4.63, *p* < 0.05), Age→IADL disability (Δχ^2^ = 4.31, *p* < 0.05), Age→DP13 (χ^2^ = 4.25, *p* < 0.05), Age→LF13 (Δχ^2^ = 3.99, *p* < 0.05), IADL disability→LF13 (Δχ^2^ = 4.43, *p* < 0.05), DP13→DP15 (Δχ^2^ = 4.09, *p* < 0.05), and DP13→LF15 (Δχ^2^ = 4.43, *p* < 0.05), LF13→LF15 (Δχ^2^ = 3.85, *p* < 0.05). ADL, activities of daily living; IADL, instrumental activities of daily living; DP13, depression in 2013–2014; DP15, depression in 2015–2016; LF13, life satisfaction in 2013–2014; LF15, life satisfaction in 2015–2016. ***p < 0.001.

In order to realize the possible gender differences, the structural equation model among the men and women samples was tested, respectively (Figure 4). The goodness-of-fit indices (Table 4) show that the models of both men and women were both acceptable. The structural equation model results were quite the same with the results in the whole sample. Notably, the parameter of ADL disability→LF13 was only significant among women (β = −0.24, *p* < 0.001), whereas the parameter of IADL disability→LF13 was only significant among men (β = −0.15, *p* < 0.001). That is to say, difficulties in ADL (e.g., dressing, bathing, eating, getting into or out of bed, using the toilet, and controlling urination and defecation) were more likely to lower women’s life satisfaction. Nevertheless, difficulties in IADL (e.g., performing household chores, preparing hot meals, grocery shopping, making phone calls, taking medications, or managing money) were more likely to lower men’s life satisfaction. In addition, Monte Carlo resampling was used to construct the CIs of the mediation models of the men and women samples. The results are shown in Table 5. In the “Age→ADL disability→LF13” path and “ADL disability→LF13→DP15” path, the 95% CI contained 0 in the men sample and did not contain 0 in the women sample. However, in the “Age→IADL disability→LF13” and “IADL disability→LF13→DP15” paths, the 95% CI did not contain 0 in the men sample and contained 0 in the women sample.

**TABLE 4:** The goodness-of-fit indices for men and women mediating model.

Model	χ^2^	df	CFI	NFI	TLI	RMSEA
Men	2321.31	550	0.99	0.99	0.99	0.031
Women	3113.44	550	0.99	0.99	0.99	0.032

**TABLE 5:** Mediating effects in different gender mediating models.

Path	Men		Women	
	Indirect effect	95% CI	Indirect effect	95% CI
Age→ADL disability→DP13	0.034	[0.026, 0.041]	0.050	[0.040, 0.060]
Age→ADL disability→LF13	–0.008	[–0.019, 0.002]	–0.038	[–0.051, –0.022]
Age→IADL disability→DP13	0.025	[0.015, 0.035]	0.041	[0.031, 0.053]
Age→IADL disability→LF13	–0.029	[–0.044, –0.013]	–0.007	[–0.023, 0.009]
Age→DP13→LF15	0.025	[0.017, 0.034]	0.025	[0.018, 0.033]
Age→LF13→DP15	–0.012	[−0.019, −0.006]	–0.009	[−0.011, −0.004]
ADL disability→DP13→LF15	–0.070	[−0.094, −0.049]	–0.065	[−0.084, −0.048]
ADL disability→LF13→DP15	0.005	[–0.001, 0.013]	0.012	[0.005, 0.020]
IADL disability→DP13→LF15	–0.033	[−0.049, −0.018]	–0.038	[−0.051, −0.026]
IADL disability→LF13→DP15	0.011	[0.004, 0.019]	0.002	[–0.002, 0.006]

*ADL, activities of daily living; IADL, instrumental activities of daily living; DP13, depression in 2013–2014; DP15, depression in 2015–2016; LF13, life satisfaction in 2013–2014; LF15, life satisfaction in 2015–2016.*

Furthermore, we used multigroup comparison analysis to clarify the gender difference among relationships between each two latent variables (refer to the note in Figure 4). It was found that the paths of Age→ADL disability (Δχ^2^ = 4.63, *p* < 0.05), Age→IADL disability (Δχ^2^ = 4.31, *p* < 0.05), Age→DP13 (Δχ^2^ = 4.25, *p* < 0.05), Age→LF13 (Δχ^2^ = 3.99, *p* < 0.05), IADL disability→LF13 (Δχ^2^ = 4.43, *p* < 0.05), DP13→DP15 (Δχ^2^ = 4.09, *p* < 0.05), DP13→LF15 (Δχ^2^ = 4.43, *p* < 0.05), and LF13→LF15 (Δχ^2^ = 3.85, *p* < 0.05) had significant gender differences. The path coefficients of Age→ADL disability in men and women samples were 0.12 (*p* < 0.001) and 0.16 (*p* < 0.001), respectively, and the path coefficients of Age→IADL disability in men and women samples were 0.19 (*p* < 0.001) and 0.23 (*p* < 0.001), respectively. The results suggest that increasing age may lead to more ADL disability and IADL disability in women than in men. However, women experience more relief from depression and higher life satisfaction than men as they get older. The path coefficients of IADL disability→LF13 in men and women samples were −0.15 (*p* < 0.001) and −0.03 (*p* > 0.05), respectively, which showed that IADL disability reduced life satisfaction only in men. The path coefficients of DP13→DP15 in men and women samples were 0.61 (*p* < 0.001) and 0.62 (*p* < 0.001), respectively, indicating that the lasting effect of depression has an extremely significant effect on both genders. Although the coefficients of this pathway were similar in the two samples, significant gender difference still exists according to the multigroup comparison analysis. That is, the lasting effect of depression was greater in women than in men. Besides, the coefficients of DP13→LF15 were −0.25 (*p* < 0.001) and −0.21 (*p* < 0.001) in men and women samples, respectively. It declared that the early prevention of depression in men had a more significant effect on the improvement of subsequent life satisfaction than that in women. Furthermore, the lasting effect of life satisfaction was greater in women (β = 0.32, *p* < 0.001) than in men (β = 0.28, *p* < 0.001). Moreover, the path coefficient of ADL→LF13 in men and women were −0.07 (*p* > 0.05) and −0.24 (*p* < 0.001), respectively. The non-significant result in men may be due to the SE of the beta was moderately large, the impacts of ADL disability on life satisfaction (95% CI [−0.16, 0.02]) among males were diverse. However, the gender difference of the path coefficient was not statistically significant by using the multigroup comparison.

### Symptoms of Functional Disability and Depression

According to lambda values (λ > 0.85) in the structural equation model, we found that regarding ADL, middle-aged and older people with functional disability had great difficulties with dressing (λ = 1.00), bathing/showering (λ = 0.99), eating (λ = 0.90), and getting into/out of bed (λ = 0.90), while regarding IADL, they had great difficulties in performing household chores (λ = 0.98), preparing hot meals (λ = 1.00), and shopping for groceries (λ = 0.94). In addition, depression among this population was mainly characterized by feeling depressed (λ = 1.00), felt everything did was an effort (λ = 0.86), feeling lonely (λ = 0.89), and feeling unable to move on (λ = 0.93). The findings could be applied to geriatric care, quality improvement, program dissemination, and service design.

## Discussion

In this study, a mediating model was used to test the effects of age on depression and life satisfaction and the mechanisms of ADL disability and IADL disability. The results showed that depression among middle-aged people was higher than that among older people and that life satisfaction was lower than that among older people. ADL disability and IADL disability played a partial mediating role in this process, and the predictive effect of IADL disability on life satisfaction in men was significantly greater than that in women. To prove the stability of this result, age was taken as an ordinal variable, which was divided into three groups and four groups, respectively, by constructing an alternative SEM. There were only a few path coefficients that differed ±0.01 from the original SEM.

### Depression and Life Satisfaction in Middle-Aged and Older Adults

Some studies have suggested that older people have higher depression (Bergdahl et al., 2005; Solhaug et al., 2012) and lower satisfaction (Kim et al., 2018) than middle-aged people. However, another study found that the frequency of depression was higher (Trollor et al., 2007), and life satisfaction was lower among middle-aged people (An et al., 2020). In addition, a study found that the 1-year prevalence rate of depression was 7.7–9.4% among middle-aged people, and 2.6% among older adults (Kessler et al., 2010). These results show that the conclusions are still inconsistent. Our study demonstrated that depression was higher and life satisfaction was lower among middle-aged people than that among older people. Among middle-aged people, although midlife introduces psychosocial resources for physical and mental health, it also carries the risk of depression (Ellermann and Reed, 2001). Middle age is a time when people balance multiple roles and responsibilities in various areas of life, such as work and family (Lachman, 2004). Social responsibility may lead to a greater burden and pressure for middle-aged people. As a result, middle-aged people may be more severely affected by the demands and increased responsibilities of midlife, leading to more negative emotions. Therefore, middle-aged people have a higher risk of mental health and should receive more attention.

### Mediating Effects of Activities of Daily Living Disability and Instrumental Activities of Daily Living Disability

The functional disability plays an important mediating role in the relationship between age and depression/life satisfaction. With increasing age, ADL disability and IADL disability may exist, which can affect depression (Ahn and Kim, 2015; Ahmad et al., 2020) and life satisfaction (Enkvist et al., 2012) simultaneously. Additionally, ADL disability has a greater effect on this process than IADL disability. The results confirm the previous studies showing that functional disability may be a crucial risk factor for depression in middle-aged and older persons (Qiu et al., 2020). Furthermore, the risk of functional disability in the older age group was higher than that in the younger age groups. The main reason for this finding may be that with increasing age, the functions of the body tissues and organs are weakened, immunity is reduced, and the ability to resist adverse external factors is weakened. Therefore, it is especially important to improve the daily living ability of older persons, delay the decline of their functional disability, reduce their depression, and maintain their life satisfaction. To prevent functional disability, it is recommended that effective health literacy education and assistance focusing on the top four difficulties with ADL (i.e., dressing, bathing or showering, eating, and getting into/out of bed) be offered. Regarding IADL, the difficulties of older people in performing household chores, preparing hot meals, and shopping for groceries could be solved with intelligent equipment, door-to-door delivery, shopping assistance, and other measures.

### Depression and Life Satisfaction May Affect Each Other and Have Enduring Effects

Previous studies (Sok, 2010; Yoo et al., 2016) have found that life satisfaction is associated with depression, but the causal relationship is unclear. Our structural equation model further showed that depression and life satisfaction may be cross affected by each other and have enduring effects. That is, the influence of previous depression (in 2013–2014) on subsequent life satisfaction (in 2015–2016) was higher than that of previous life satisfaction (in 2013–2014) on subsequent depression (in 2015–2016). The standardized coefficients were −0.18 and −0.07, respectively. Furthermore, depression among middle-aged and older adults may have a lasting effect, which is consistent with a previous study (Lee et al., 2020). Our findings clarify that reducing or preventing depression as early as possible may be a more effective approach to preventing depression among middle-aged and elderly people than improving life satisfaction. The standardized coefficients were 0.60 and −0.07, respectively.

As mentation to maintain long-term life satisfaction among middle-aged and elderly people, enhancing their current life satisfaction and preventing depression are also effective ways. Furthermore, functional disability may increase depression and lead to a decrease in life satisfaction. Prevention is better than cure, and we recommend that avoiding functional disability could result in reduced depression and improved life satisfaction.

### Gender Differences

Regarding ADL disability, IADL disability, depression, and life satisfaction in middle-aged and older adults, the results showed that women had higher levels of IADL disability and perceived higher depression and lower life satisfaction than men. However, there was no gender difference in ADL disability, which is consistent with the conclusion of a previous study (Sato et al., 2001). The gender is an important factor in studies of functional disability, depression, and life satisfaction. Fertility, hormonal, and other physiological differences lead to different health risks between men and women. In addition, work, family, and lifestyle roles differ between women and men. The traditional role of caregivers and family workers in the family has a significant detrimental impact on the health status of older women (Zhang et al., 2005). But men’s mental health also needs to be taken seriously. Our findings further demonstrate that gender differences exist on some pathways. Life satisfaction in men was influenced by IADL disability, whereas, in women, it was influenced by ADL disability.

### Limitations

Some limitations to this study warrant consideration. First, the associations among age, functional ability, and depressive symptoms/life satisfaction are cross-sectional in the study and can be further validated using longitudinal data in the future. Second, the correlation between age and depression was significant but small. Whether age is weakly correlated with depression or affected by sample size requires more specific studies to clarify. Third, since the information was gathered from the participants in the study, self-report/recall bias may have existed. However, it is not easy to achieve continued participation among cohorts of middle-aged and older people in a cohort study, and the sample size should not be ignored. As a result, our findings with acceptable goodness-of-fit indices deserve paying more attention.

## Conclusion

Depression in middle-aged people should be given closer attention. Depression and life satisfaction could affect each other and have enduring effects. Functional disability was an important mediator of depression and life satisfaction. Avoiding or improving functional disability may be an effective way to improve life satisfaction and reduce the level of depression in middle-aged and older persons. For the prevention of functional disability and depression and improvement in life satisfaction, gender differences need to be considered.

## Data Availability Statement

The original contributions presented in the study are included in the article/supplementary material, further inquiries can be directed to the corresponding authors.

## Ethics Statement

The studies involving human participants were reviewed and approved by the Ethical Review Committee of Peking University. The patients/participants provided their written informed consent to participate in this study.

## Author Contributions

AL and Y-CC designed the study, analyzed the results, and drafted and revised the manuscript. SL designed the study and drafted and revised the manuscript. MC and SH drafted and revised the manuscript. C-YL and DW analyzed the results and revised the manuscript. All authors read and approved the final article.

## Conflict of Interest

The authors declare that the research was conducted in the absence of any commercial or financial relationships that could be construed as a potential conflict of interest.

## Publisher’s Note

All claims expressed in this article are solely those of the authors and do not necessarily represent those of their affiliated organizations, or those of the publisher, the editors and the reviewers. Any product that may be evaluated in this article, or claim that may be made by its manufacturer, is not guaranteed or endorsed by the publisher.

## Abbreviations

ADL: activities of daily living
IADL: instrumental activities of daily living
WHO: World Health Organization
DP: depression
LF: life satisfaction
DP13: depression in 2013–2014
DP15: depression in 2015–2016
LF13: life satisfaction in 2013–2014
LF15: life satisfaction in 2015–2016
CHARLS: China Health and Retirement Longitudinal Study
CES-D: The Center for Epidemiological Studies Depression Scale
CFA: confirmatory factor analysis
*M*: mean
SD: standard deviation
DE: direct effect
IE: indirect effect
TE: total effect
SEM: structural equation modeling.
